# Generation and Evaluation of Isogenic iPSC as a Source of Cell Replacement Therapies in Patients with Kearns Sayre Syndrome

**DOI:** 10.3390/cells10030568

**Published:** 2021-03-05

**Authors:** Glen Lester Sequiera, Abhay Srivastava, Keshav Narayan Alagarsamy, Cheryl Rockman-Greenberg, Sanjiv Dhingra

**Affiliations:** 1 St. Boniface Hospital Albrechtsen Research Centre, Regenerative Medicine Program, Department of Physiology and Pathophysiology, Rady Faculty of Health Sciences, Institute of Cardiovascular Sciences, Max Rady College of Medicine, University of Manitoba, Winnipeg, MB R2H 2A6, Canada; gsequiera@sbrc.ca (G.L.S.); asrivastava@sbrc.ca (A.S.); kalagarsamy@sbrc.ca (K.N.A.); 2 Department of Pediatrics and Child Health, Rady Faculty of Health Sciences, Max Rady College of Medicine, University of Manitoba, Winnipeg, MB R3E 3P4, Canada; cgreenberg@hsc.mb.ca

**Keywords:** Kearns Sayre syndrome, mitochondria, iPSC, stem cells

## Abstract

Kearns Sayre syndrome (KSS) is mitochondrial multisystem disorder with no proven effective treatment. The underlying cause for multisystem involvement is the energy deficit resulting from the load of mutant mitochondrial DNA (mtDNA), which manifests as loss of cells and tissue dysfunction. Therefore, functional organ or cellular replacement provides a promising avenue as a therapeutic option. Patient-specific induced pluripotent stem cells (iPSC) have become a handy tool to create personalized cell -based therapies. iPSC are capable of self-renewal, differentiation into all types of body cells including cardiomyocytes (CM) and neural progenitor cells (NPC). In KSS patients, mutations in mtDNA are largely found in the muscle tissue and are predominantly absent in the blood cells. Therefore, we conceptualized that peripheral blood mononuclear cells (PBMNC) from KSS patients can be reprogrammed to generate mutation free, patient specific iPSC lines that can be used as isogenic source of cell replacement therapies to treat affected organs. In the current study we generated iPSC lines from two female patients with clinical diagnosis of classic KSS. Our data demonstrate that iPSC from these KSS patients showed normal differentiation potential toward CM, NPC and fibroblasts without any mtDNA deletions over passages. Next, we also found that functional studies including ATP production, reactive oxygen species generation, lactate accumulation and mitochondrial membrane potential in iPSC, CM, NPC and fibroblasts of these KSS patients were not different from respective cells from healthy controls. PBMNCs from these KSS patients in the current study did not reproduce mtDNA mutations which were present in muscle biopsies. Furthermore, we demonstrate for the first time that this phenomenon provides opportunities to create isogenic mutation free iPSC with absent or very low level of expression of mtDNA deletion which can be banked for future cell replacement therapies in these patients as the disease progresses.

## 1. Introduction

Inborn mitochondrial disorders are highly variable multisystem disorders which are caused by mutations in mitochondrial DNA (mtDNA) leading to dysfunction in mitochondrial respiratory chain complexes, adversely affecting energy production in cells [1]. This leads to impaired development and functioning of many cell types of the body [2]. Human mtDNA is a circular molecule of 16,569 bp containing 37 genes including 2 ribosomal RNAs, 22 tRNAs and 13 genes encoding subunits of the respiratory chain. Cells contain thousands of mtDNA molecules and when mutations exist, cells usually harbor both wild type (wt) and mutant mtDNA molecules, a phenomenon known as heteroplasmy. Kearns Sayre syndrome (KSS) is one such multisystem disorder, the disease progresses slowly, over decades, with new symptoms appearing and previous symptoms slowly worsening. The large mtDNA deletions in KSS often encompass the subunits of mtDNA involved in the oxidative phosphorylation pathway [3]. Symptoms only appear if the proportion of abnormal DNA reaches a certain threshold. The treatment options for patients suffering with KSS are very limited. The major factors behind this are multi-systemic nature of disease progression and the variability in disease manifestation in different patients owing to differences in mutation load and heteroplasmy in various organs [4]. The traditional treatment strategies are based on broad “applicable-to-all” approaches. This involves administration of a treatment which non-specifically targets all the patients and all the organs within them. Such approaches are usually aimed to correcting the electron transport chain (ETC) defect by supplementing intermediates such as co-enzyme Q10, cofactors such as riboflavin or nicotinamide riboside to engineer higher production of the cofactor nicotinamide adenine dinucleotide [5,6]. These approaches have been found to be generally ineffective [7].

However, more recently, the emerging therapies are tailored toward patient-specific modalities after understanding the distinct defective mechanism. For example, supplementation of patient-specific deficient enzymes or substrates, gene therapy and cell replacement therapies (CRTs) are some of the approaches which are being explored recently for many inborn disorders. In fact, the enzyme replacement therapy or substrate reduction therapy are approved for many lysosomal storage disorders, e.g., Gaucher disease, Fabry disease, Pompe disease and the mucopolysaccharidoses [8]. These modalities have demonstrated generally good results, adapted to the precise mutation and the organs. The cell replacement therapies involve transplantation of stem cells to injured or impaired organs. In fact stem cells based therapies now have potential to treat several degenerative diseases and inborn disorders [9]. Stem cells are able to differentiate into multiple cell types in the body and replace dysfunctional or dead cells to revive tissue or organ function. In this regard, patient -specific induced pluripotent stem cell (iPSC) lines allow to develop personalized therapies in an individual- specific manner. Additionally, iPSC offer several advantages including ability to differentiate into multiple cell types, therefore iPSC are an inexhaustive source of different cells. Further, there are no ethical issues involved with iPSC-based therapies.

In patients with KSS the multi-organ involvement includes mainly the central nervous system, heart, muscle, eye, endocrine system and kidney. The signs include cerebellar ataxia, cardiac conduction defects, cardiomyopathies, ptosis, pigmentary retinopathy, complete ophthalmoplegia, skeletal myopathy, endocrinopathies and renal impairment [10,11]. As KSS slowly progresses, prognosis for the affected individuals is determined by the type and extent of organs involved, which increases with time. The energy deficit manifests as loss of cells and tissue function compromise. Therefore, functional organ or cellular replacement provides a promising avenue to stop or reverse the disease progression. In this regard, iPSC derived differentiated cells including cardiomyocytes, neural cells, fibroblasts, retinal cells and renal cells have been employed to treat or prevent disease progression in case of different disorders including cardiovascular, central nervous system disorders, retinopathies and renal disorders [12,13,14,15]. However, iPSC-based technologies have not yet been used as a source of cell replacement personalized therapies to treat the multi-organ complications in KSS patients.

The mtDNA deletions in patients with KSS are largely found in the muscle tissue and are predominantly absent in the blood cells [16]. Therefore, we hypothesized that this phenomenon provides an opportunity to selectively take blood cells (which are free of mutations) from KSS patients and reprogram these to generate normal iPSC. Furthermore, these patient-derived “mutation free” isogenic iPSC can be differentiated toward cardiomyocytes, neurons and other lineages and can be employed as a source of cell replacement personalized therapies in a patient specific manner. Therefore, in the current proof of concept study, we derived blood from two female patients with clinical diagnosis of classic KSS. The iPSC developed from these patients were differentiated into cardiomyocytes (CM), neural progenitor cells (NPC) and fibroblasts. Thereafter, iPSC and differentiated cells were tested for the presence of mutations and metabolic changes normally observed in affected cells from KSS patients. To the best of our knowledge this is the first study demonstrating the generation of isogenic “mutation free” iPSC lines from KSS patients which can be banked for future cell replacement therapies in these patients as the disease progresses.

## 2. Materials and Methods

### 2.1. Collection of Blood Samples

Blood (10 mL) was collected from two clinically diagnosed female patients with Kearne-Sayre syndrome (KSS1 and KSS2) and two age and sex matched healthy individuals (controls) as well as a positive control (patient with Leigh syndrome harboring compound heterozygous mutations in the NDUFV1 gene). The age of patients at the time of blood sample collection for iPSC generation was, 50 year (KSS1) and 65 year (KSS2). Prior to blood collection, written informed consents from all the patients and healthy individuals (controls) were obtained. The peripheral blood mononuclear cells (PBMNC) were extracted using the Lympholyte-H kit (Cedarlane, Burlington, ON, Canada) according to the manufacturer’s protocol. All the protocols and procedures were approved by the University of Manitoba’s Health Research Ethics Board (REB # B2015:025).

### 2.2. mtDNA Analysis of the Patients

The molecular studies in both patients (KSS1 and KSS2) were carried out at the Biochemical Disease Laboratory, Children’s & Women’s Health Centre of British Columbia, Vancouver, BC, Canada. Both the patients underwent quadricep muscle biopsies in 1994 and 2003, respectively, for confirmatory diagnostic testing. The mitochondrial DNA analysis was performed on muscle mitochondria isolated from muscle biopsies by previously described procedures [17]. Briefly, for patient KSS1, conventional PCR was performed using forward and reverse oligonucleotide primers known to detect the “common deletion” of 4.9 kb [17]. PCR products were separated on 0.8% agarose gels, and the data were confirmed using Southern blotting. For patient KSS2 long range PCR which amplifies DNA over a large range was employed using a Displacement (D)-loop primer pair. The mtDNA D-loop occurs in the main non-coding “control” region of the mitochondrial DNA genome. The forward primer ended at nucleotide 15,900 and reverse primer ended at nucleotide 15,400 in the D-loop.

The DNA testing of positive control (patient with Leigh syndrome) for mitochondrial disorders using a Mitochondrial Encephalopathy/Leigh syndrome nuclear gene panel (GENEDx, Gaithersburg, MD, USA) revealed 2 variants in the *NDUFV1* gene. The first pathogenic variant is c.529dupT(p.Y177Lfs*z2) and the second is c.640G>A(p.E214k).

### 2.3. Generation and Maintenance of iPSC

iPSC were generated by reprogramming PBMNC using a commercial kit (CytoTune^®^-iPS 2.0 Sendai Reprogramming Kit, Thermo Fisher Scientific, Winnipeg, MB, Canada). Briefly, PBMNC (4 × 10^5^ cells) were cultured in 6-well plates. After 24 h, the cells were transduced with Sendai virus, and after 4 days of virus transduction, PBMNC were plated onto Geltrex (Thermo Fisher Scientific)-coated plates and cultured in a mixture of PBMNC medium (Thermo Fisher Scientific) and TeSR™-E8™ (Stemcell Technologies Inc., Vancouver, BC, Canada) medium at a ratio of 1:1. When cells started attaching to the Geltrex, the culture medium was replaced with TeSR™-E8™. After a successful adaptation to feeder-free conditions at least three iPSC colonies from each patient and healthy controls were expanded in TeSR™-E8™ medium using manual gridding protocol. Mycoplasma testing was carried out using NucBlue Live Cell Stain (R37605, Thermo Fisher Scientific; data not shown)

### 2.4. Evaluation of Pluripotency of iPSC

The generated iPSC were immunostained with pluripotency markers- OCT4A, SOX2, NANOG, SSEA4, TRA-1-60, TRA-1-81 using StemLight Pluripotency Antibody Kit (Cell Signaling, Danvers, MA, USA). Briefly, the cells were fixed with 4% paraformaldehyde and permeabilized with ice-cold methanol for 10 min at −20 °C and then rinsed with phosphate buffer saline (PBS) for 5 min. After blocking with 5% BSA (containing 0.3% Triton X-100) for 60 min, the iPSC were stained with primary antibody (1:500) for 12 h at 4 °C, this was followed by fluorochrome-conjugated secondary antibody (1:1000) for 2 h at room temperature. The cells were mounted using Prolong Antifade reagent containing DAPI. The iPSC were finally rinsed with PBS and then examined microscopically (Cytation 5, Biotek Instruments, Winooski, VT, USA).

### 2.5. Assessment of Tri-Lineage Differentiation Potential of iPSC

At day 6 iPSC colonies were treated with 5 U/mL of dispase for 5 min this was followed by gently lifting the whole colonies. The colonies were gently transferred to Nunclon Spehra Low attachment dishes. The suspended iPSC colonies were supplemented with embryoid bodies (EB) medium containing DMEM, 10% FBS, nonessential amino acids, β-mercaptoethanol, l-glutamine, and penicillin/streptomycin (Thermo Fisher Scientific). The iPSC colonies formed solid balls after 1 day of suspended culture. These EB were cultured in suspension for 8 days and subsequently plated onto gelatin coated dishes on day 8. The trilineage differentiation potential of EB was carried out using STEMdiff™ Trilineage Differentiation Kit (Stemcell Technologies Inc.). 

### 2.6. Differentiation of iPSC to Fibroblasts

Fibroblasts were obtained through spontaneous differentiation of the EB. The manually dissected fibroblast layers were expanded in the culture and then characterized using HSP47 (Cat#AB77609, Abcam, Cambridge, UK) and FSP (Cat#AB11333, Abcam). The cells which were >98% positive for HSP47 and FSP were utilized for further studies. 

### 2.7. Differentiation of iPSC to NPC

For NPC differentiation, the EB (once formed) were treated with SB 431,542 (Torcis, Toronto, ON, Canada), a TGF-beta/Smad inhibitor for 2 days in the EB medium. On day 5, the suspended EB were plated on dishes coated with polyornithine. Thereafter, the EB were observed for the classic neural rosette structures. These rosettes are apparent around day 7–10. After visual confirmation, the rosettes were excised and allowed for selective expansion on polyornithine coated plates supplemented with Neurocell medium (Wisent Bioproducts, Montreal, QC, Canada). The characterization of NPC was performed by immunostaining the cells with SOX2 (Cat#4900S, Cell Signaling technologies) and NESTIN (Cat#sc-23927, SantaCruz Biotech, Dallas, TX, USA).

### 2.8. Differentiation of iPSC to Cardiomyocytes

Single cell culture was achieved by subjecting iPSC (>p20) to versene treatment. The cells were plated again and allowed to grow to >75% confluency. The medium was changed to cardiac differentiation medium—RPMI 1640 (Thermo Fisher Scientific), 500 μg × mL^−1^ Oryza sativa–derived recombinant human albumin (Sigma-Aldrich, Oakville, ON, Canada), and 213 μg × mL^−1^ L-ascorbic acid 2-phosphate (Sigma-Aldrich), the medium was replenished at 48 h. The small molecules were added to initiate cardiac differentiation in the following sequence—days 0–2 (CHIR99021, 6 μM) and days 2–4 (Wnt-C59, 2 μM). On day 4, cardiac differentiation medium without any small molecules was used to maintain the differentiation. The day 7 onwards, the cultures were observed for contracting cells and the batch with cardiac differentiation efficiency over 90% was used for further experiments. Immunostaining was carried out to ensure cardiac lineage using cardiac troponin (CTNT) (Cat#AB8295, Abcam), Homeobox protein NKX-2.5 (Cat#8792S, Cell signaling technologies).

### 2.9. ATP Measurement

The ATP levels in iPSC, fibroblasts, NPC and CM were quantified using a luminescent ATP detection assay kit (Abcam, Cat#ab113849) according to the manufacturer’s directions. The cells (1 × 10^5^ cells/well) were plated and cultured in a 96-well plate using specific medium for each cell type. Thereafter, the plate was centrifuged at 1000 rpm for 5 min to pellet floating cells. ATP standards were processed within the same plate to establish reference points. The Cytation 5 plate reader (Biotek, Instruments) was used for quantification of plates.

### 2.10. Measurement of Lactate Levels

The L-Lactate assay kit (Cat# ab65330, Abcam) was used to measure total lactate concentration in iPSC, fibroblasts, NPC and CM. The cell lysates were prepared using manufacturer-provided reagents and lactate levels were measured according to the manufacturer’s directions. The optical density was read using Cytation 5 plate reader (Biotek, Instruments) at a wavelength of 570 nm. 

### 2.11. Measurement of Mitochondrial Membrane Potential (Ψm)

The tetramethylrhodamine ethyl ester (TMRE) assay was employed to measure Ψm. Briefly, 1 nM of TMRE in medium was added to iPSC, fibroblasts, NPC and CM for 30 min at room temperature. Then, 50 μM of FCCP (carbonyl cyanide 4-(trifluoromethoxy) phenylhydrazone) was also added to a set of all cell types as a negative control. The fluorescence of the wells was read using Cytation 5 plate reader (Biotek, Instruments) at excitation wavelength 549 nm and emission wavelength 575 nm.

### 2.12. Assessment of Cellular Growth

To measure cellular growth, 1 × 10^4^ cells per well were plated in 96-well plates. The cells were then incubated with a stain -CytoPainter (Cat #ab176735, Abcam). The cell number after 2 days of culture was counted. The data were calculated as ratio of day 2 count vs. initial cell number (1 × 10^4^). 

### 2.13. Measurement of Reactive Oxygen Species Generation

Reactive oxygen species (ROS) generation was measured in iPSC, fibroblasts, NPC and CM using a dye 2′,7′-dichlorodihydrofluorescein diacetate (H2DCFDA). Briefly, the cells (1 × 10^6^) were plated in a 96-well plate, after washing with PBS, 5 μM of H2DCFDA was added. This was accompanied by addition of 0.5 μg/mL Hoechst to the cells. The fluorescence signal was recorded using Cytation 5 plate reader (Biotek, Instruments). 

### 2.14. qPCR Analyses

Quantitative PCR was performed to measure mRNA expression of *POU5F1* (5′-GGAAGGAATTGGGAACACAAAGG/3′-AACTTCACCTTCCCTCCAACCA), *Tra-1-60* (5′ CAACCCGGCCCAAGATAAGT/3′-GGCAGGGAGCTTAGTGTGAA), *Sox17* (5′-GAGCCAAGGGCGAGTCCCGTA/3′-CCTTCCACGACTTGCCCAGCAT), *Foxa2* (5′-CCCCTGAGTTGGCGGTGGT/3′-TTGCTCACGGAAGAGTAG), *Brachury T* (5′-ACCCAGTTCATAGCGGTGAC/3′-CCATTGGGAGTACCCAGGTT), *NCam1* (5′-AACAAAGCATGATGGGTGAA/3′-GTCTGTGGTGTTGGAAATGC), *OTX2* (5′-CAAAGTGAGACCTGCCAAAAAGA/3′-TGGACAAGGGATCTGACAGTG), *Pax6* (5′-ATGGGCGGAGTTATGATACCTAC/3′-GGAACTTGAACTGGAACTGACA), *MT-CO3* (5′-TTTCCGACGGCATCTACGG/3′-TACAAAATGCCAGTATCAGGCG), *16S rRNA* (5′-TTTACGACCTCGATGTTGGATCAG/3′-CCTTTCGTACAGGGAGGAATTTG) and *HPRT1* (5′-CACGGCTGTGCTAGTTCAGTA/3′-TCGACAAGCCCAGAAACTTGT) (Supplementary Table S1) using PowerUp SYBR Green Master Mix (Thermo Fisher Scientific). The reaction was performed in Applied Biosystems^®^ QuantStudio™ 5 Flex Real-Time PCR System (Thermo Fisher Scientific). The data were analyzed using the QuantStudio™ software (Thermo Fisher Scientific) and relative gene expression was determined using the 2-∆∆Ct method using a housekeeping gene (*RPLPO*: 5′-CACTGGCTGAAAAGGTCAAGG/3′-GACTTGGTGTGAGGGGCTTA).

### 2.15. Statistical Analysis:

Data are presented as mean ± SD. The significance of the data was evaluated by Student’s *t*-test or two-way ANOVA with Bonferroni post hoc test. *p* < 0.05 was considered statistically significant. Unless otherwise stated, ≥3 independent experiments were used for all assays and displayed figures are representative.

## 3. Results

### 3.1. Clinical and Molecular Characterization of the Patients

**Figure 1:**
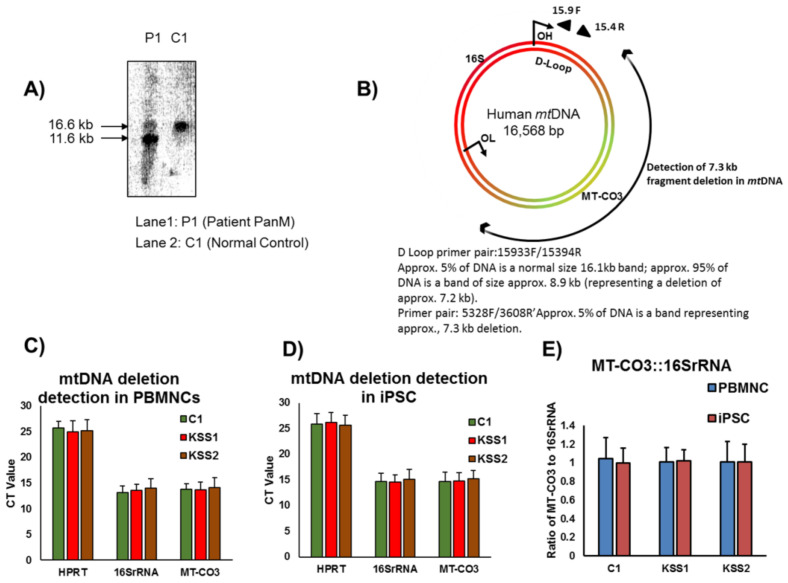
Clinical characterization of patients and PCR analysis of peripheral blood mononuclear cells (PBMNC) and induced pluripotent stem cells (iPSC). (**A**) Clinical report of Kearns Sayre syndrome (KSS) patient labelled as KSS1. Southern blotting of mitochondrial DNA (mtDNA) from muscle biopsy denotes a ~5 kb deletion leading to two bands. (**B**) Clinical diagnosis report of the patient labelled as KSS2 denotes a deletion of ~7.3 kb in the mtDNA from the muscle biopsy. (**C**,**D**) mtDNA deletion detection was conducted by carrying out PCR reaction using a set of 3 primers specific to regions in-genetic DNA (Hypoxanthine Phosphoribosyltransferase 1 (*HPRT)*), possible deletion site on mtDNA (Mitochondrial complex III (*MT-CO3)*) and non-affected site on mtDNA (16S Ribosomal RNA (*16S rRNA)*). No deletion was observed in either the PBMNC (**C**) or, respectively, derived iPSC (**D**). The deletions if present would have been denoted by increase in the Ct value for *MT-CO3* gene. (**E**) Ratio of the Ct values of the *MT-CO3* to the Ct values of *16S rRNA* were calculated for the control, KSS1 and KSS2 samples (PBMNC and iPSC). The ratios denote absence of any mtDNA deletion in the samples. *n* = 3, each experiment (in panels C, D and E) was repeated 3 times.

Both patients (KSS1 and KSS2) manifested signs and symptoms of classic KSS with variable degrees of slowly progressive muscle weakness, ptosis and complete ophthalmoplegia and hearing loss. The mtDNA analysis of KSS1 patient indicated a ~5 kb deletion confirmed on Southern blotting with ~80% of mtDNA deleted in the “major arc” of the mtDNA molecule. The KSS2 patient was found to have a mtDNA deletion of ~7.3 kb in the muscle biopsy. The Figure 1A,B are the representations of clinical reports of these two patients.

### 3.2. Peripheral Blood Mononuclear Cells from Patients Did Not Reproduce Mutations

Before proceeding further with the development of iPSC from patient samples as a source of cell replacement therapies, we screened peripheral blood mononuclear cells (PBMNC) from the KSS patients for presence of mutations using real time PCR. Our data confirmed the absence of any mtDNA deletions in PBMNC from both the patients (Figure 1C). The PBMNC from each patient and healthy controls were reprogrammed to iPSC as described in the next section and evaluated for presence of mutations. The outcome of PCR analysis confirmed that there were no mtDNA deletions in the iPSC lines established from both the patients (Figure 1D). On the other hand we were able to confirm the presence of mutation in PBMNC and iPSC of positive control (Leigh syndrome patient) as presented in our recent study [18]. To better represent the presence or absence of mtDNA deletion, the ratio of MT-CO3: 16S rRNA for the cells was calculated. In case of PBMNC, the ratio was: 1.04 for control, 1.01 for KSS1 and 1.01 for KSS2. Similarly, for the iPSC the ratio was calculated to be: 0.99 for control, 1.02 for KSS1 and 1.01 for KSS2 (Figure 1E).

### 3.3. Reprogramming of Patient Derived PBMNC to iPSC and Characterization

**Figure 2:**
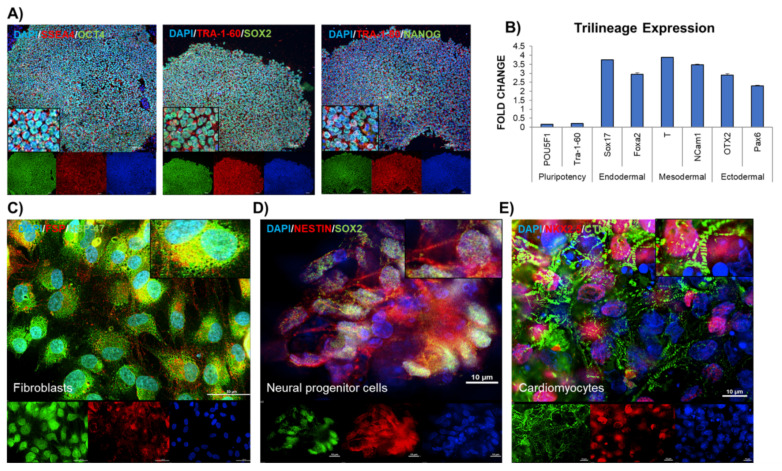
Characterization of pluripotency and differentiation potential of induced pluripotent stem cells (iPSC). (**A**) Characterization of the pluripotency was carried out by immunostaining of the pluripotency markers—SSEA4, TRA-1-60, TRA-1-81 in red and OCT4, SOX2, NANOG in green. (**B**) Assessment of tri-lineage differentiation potential of iPSC was carried out by initiation of spontaneous differentiation by embryoid body generation. The embryoid bodies in suspension cultures differentiate into cells from all the 3 germ layers. These were detected by qPCR by targeting early lineage specific markers—*POU5F1* and *TRA-1-60* (pluripotency), *SOX17* and *FOXA2* (Endodermal), *BRACHURY T* or *T* and *NCAM1* (Mesodermal), and *OTX2* and *PAX6* (Ectodermal). (**C**-**E**) The pluripotency was also characterized by cell specific differentiation, (**C**) Fibroblasts (immunostained for HSP47 (green) and FSP (red)), (**D**) Neural progenitors (immunostained for NESTIN (red) and SOX2 (green)) and (**E**) Cardiomyocytes (immunostained for NKX2.5 (red) and cTNT (green)), Nucleus is blue with Dapi. *n* = 3, each experiment was repeated 3–4 times.

The PBMNC derived from blood collected from patients and healthy individuals as well as positive control were reprogrammed to iPSC using a commercial kit (Thermo Fisher Scientific). The iPSC were characterized using pluripotency markers, our data showed the presence of OCT4, NANOG, SOX2, SSEA4, TRA-1-81 and TRA-1-60 in iPSC (Figure 2A).

To verify the functional pluripotency of iPSC, the embryoid bodies (EB) were formed and assessed on day 12 for trilineage differentiation potential toward mesoderm, endoderm and ectoderm (Figure 2B). The expression of *SOX17*, *FOXA2* (endoderm), *BRACHURY T* or *T* and *NCAM1* (mesoderm) and *OTX2* and *PAX6* (ectoderm) registered a significant increase in EB. Further, the pluripotency validation was also established by directed differentiation towards fibroblasts, neural and cardiac lineage (Figure 2C–E). The presence of karyotypic abnormalities in iPSC was investigated at intervals of 20–25 passages using a commercial kit (StemCell Technologies). None of the iPSC lines generated in the study reported any karyotypic abnormalities (Supplementary Figure S1).

### 3.4. Patient PBMNC Derived iPSC Show Normal Differentiation Potential 

**Figure 3:**
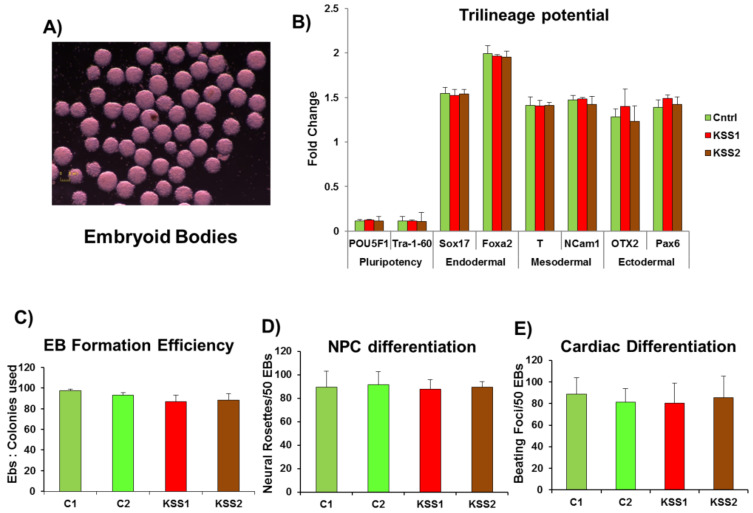
Assessment of trilineage and directed differentiation potential of induced pluripotent stem cells (iPSC). (**A**) Embryoid body suspension cultures showed uniform cellular clusters; (**B**) Trilineage differentiation potential was assessed through qPCR analysis for both (Kearns Sayre syndrome patients) KSS1 and KSS2 as well as healthy control PBMNC derived iPSC clones. (**C**) Embryoid body formation efficiency across controls and patients (KSS1 and KSS2) iPSC clones was found to be equivalent and consistent over passages; (**D**) Directed differentiation of iPSC to neural progenitor cells was carried out. (**E**) Directed differentiation of iPSC to cardiomyocytes was carried out. The differentiation potential of KSS1 and KSS2 peripheral blood mononuclear cells (PBMNC) derived iPSC clones toward neural progenitor cells and cardiomyocytes was comparable to control iPSC. *n* = 3, each experiment was repeated 3–4 times.

The embryoid bodies (EB) formation is a three-dimensional aggregate system wherein stochastic differentiation of the pluripotent stem cells into cells of all the three lineages; endoderm, mesoderm and ectoderm, defines multi-differentiation capabilities of the generated iPSC lines. The analysis of EB reveals if different cell types that may be required by the patients as the disease progresses can be achieved or might be met with difficulties. The assessment of both the patient PBMNC derived iPSC lines showed robust EB formation and multilineage differentiation potential, which was comparable to the iPSC lines generated from healthy individuals (Figure 3A–E).

Further, KSS has been regularly associated with progressive loss of memory and corresponding loss of grey and white matter which have been largely found to be underlined by loss of neural cells in the respective regions [19]. The cardiac conduction defects, on the other hand, are a key criterion for the clinical diagnosis of KSS [20]. These defects are carried over in neural and cardiac specific differentiation, which have been amply demonstrated in mtDNA deletion-based as well as genomic DNA micro-deletion based iPSC studies in case of other mitochondrial disorders [21,22,23]. In our study, both the patient PBMNC derived iPSC lines did not show any aberration either in the neural progenitor (NPC) differentiation (Figure 3D) or in the cardiac differentiation potential (Figure 3E), which was comparable to the iPSC lines generated from healthy individuals. In the next section, we assessed patient iPSC lines and iPSC derived NPC, CM and fibroblasts for metabolic changes which are normally observed in affected cells of KSS patients.

### 3.5. Patient PBMNC Derived iPSC and Their Derivatives Did Not Show Any Aberrant Deviations in the Cellular Physiology

#### 3.5.1. Adenosine Triphosphate Levels

**Figure 4:**
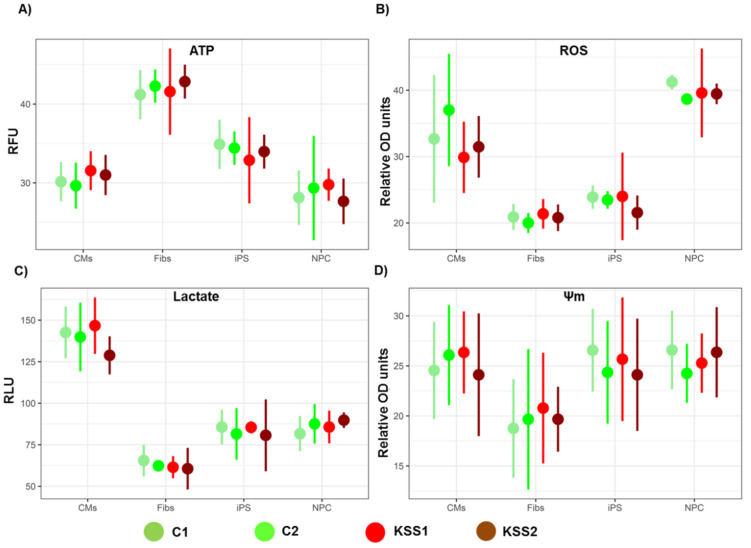
Patient peripheral blood mononuclear cells (PBMNC) derived induced pluripotent stem cells (iPSC) and differentiated cells did not show any aberrant deviations in the cellular physiology. (**A**) Total ATP production in iPSC, fibroblasts, Neural progenitor cells (NPC) and Cardiomyocytes (CM) was measured using a commercial kit. (**B**) Reactive Oxygen species (ROS) estimation was carried out in iPSC, fibroblasts, NPC and CM using a fluorescent dye 2′,7′-dichlorodihydrofluorescein diacetate (H2DCFDA). (**C**) Lactate accumulation in iPSC, fibroblast, NPC and CM was assessed using luminometric analyses. (**D**) Mitochondrial membrane potential (Ψm) in iPSC, fibroblasts, NPC and CM was estimated using Tetramethylrhodamine, ethyl ester (TMRE) assay. *n* = 3, each experiment was repeated 3–4 times.

Adenosine triphosphate (ATP) provides energy to drive different processes in the cells. The ATP levels in patients with KSS are reported to be disturbed, and alterations in intracellular ATP production is classically the main energy defect detected in these patients [24]. Interestingly, in the current study, we found no change in ATP levels in PBMNC derived iPSC of both KSS patients compared to iPSC from PBMNC of healthy individuals (Figure 4A). However, we detected a significant decrease in ATP levels in iPSC of positive control compared to healthy control (Leigh syndrome patient) (Supplementary Figure S2A).

Furthermore, ATP levels were also measured in iPSC derived fibroblasts, NPC and CM. Our data suggest that all three differentiated cell types were able to produce normal levels of ATP, as there was no significant difference detected in cells from both KSS patients and healthy individuals (Figure 4A). However, we found a significant decrease in ATP levels in all three cell types of Leigh syndrome patient compared to healthy control (positive control, Supplementary Figure S2A).

#### 3.5.2. ROS Levels

The ROS are formed as a by-product of normal metabolism of oxygen in the cells and play important role in cell signaling and homeostasis. Under stress conditions ROS levels increase dramatically and this results in damage to the cell structure. Upregulation of intracellular ROS in KSS patients is an often-registered hallmark and is usually targeted as the basis for therapy. The iPSC of the KSS patients did not record any aberrant change in ROS generation compared to the iPSC of healthy individuals (Figure 4B). Similarly, iPSC derived fibroblasts, NPC and CM also did not register a difference in ROS levels in comparison to cells from healthy individuals (Figure 4B). Interestingly we found significant increase in ROS generation in iPSC and iPSC derived fibroblasts, NPC and CM of the positive control compared to healthy control (Supplementary Figure S2B).

#### 3.5.3. Lactate Levels

In KSS patients the lactate levels are normally disturbed due to decreased ATP levels and impaired cellular respiration. This may lead to lactate accumulation which presents as lactic acidosis even at basal conditions. In literature as well as in both the patients enrolled in the current study, elevated lactate levels in the peripheral blood were infrequently documented [11]. However, in iPSC of both the patients we did not find any change in lactate levels compared to iPSC from healthy individuals (Figure 4C). Further, there were no abnormal levels of lactate accumulation in iPSC derived fibroblasts, CM and NPC of both the patients in comparison to healthy cells (Figure 4C). On the other hand, we detected a significant increase in lactate levels in iPSC and iPSC derived CM of positive control compared to healthy control (Supplementary Figure S2C).

#### 3.5.4. Mitochondrial Membrane Potential

Mitochondrial membrane potential (Ψm) is dictated by redox activity of the electron transport chain components and is a very good indicator of mitochondrial function and health. The disturbance in Ψm in KSS patients and other inborn mitochondrial disorders is often reported [25]. In the current study, we did not find any change in Ψm in iPSC of both the patients compared to iPSC from healthy individuals (Figure 4D). Additionally, there were no alterations in the levels of Ψm in iPSC derived fibroblasts; CM and NPC of both the patients in comparison to healthy cells (Figure 4D). However, we found a significant decrease in Ψm in iPSC and iPSC derived fibroblasts, NPC and CM of positive control compared to healthy control (Supplementary Figure S2D).

#### 3.5.5. Cellular Growth

**Figure 5:**
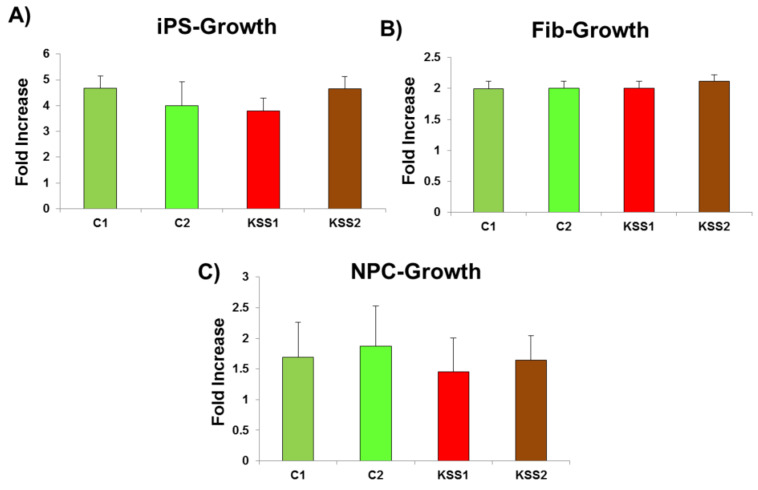
Cellular doubling analysis was carried out to assess the changes in growth potential of indiced pluripotent stem cells (iPSC) and differentiated cells from patients and healthy controls. (**A**) There was no difference in cell proliferation in iPSC of both the patients compared to iPSC from healthy individuals. (**B**,**C**) Additionally, there were no alterations in the rate of cell proliferation in iPSC derived (**B**) fibroblasts and (**C**) Neural progenitor cells (NPC) of both the patients in comparison to healthy cells. *n* = 3, each experiment was repeated 3–4 times.

Poor growth, low body mass index and loss of body weight are usually associated with the progression of KSS and other inborn mitochondrial disorders. The decrease in cellular proliferation is one of the factors underlying these clinical manifestations related to growth. Therefore, cellular growth was measured in our patient cells. We did not find any difference in cell proliferation in iPSC of both the patients compared to iPSC from healthy individuals (Figure 5A). Additionally, there were no alterations in the rate of cell proliferation in iPSC derived fibroblasts and NPC of both the patients in comparison to healthy cells (Figure 5B,C).

#### 3.5.6. Cardiomyocyte Functional Analyses 

**Figure 6:**
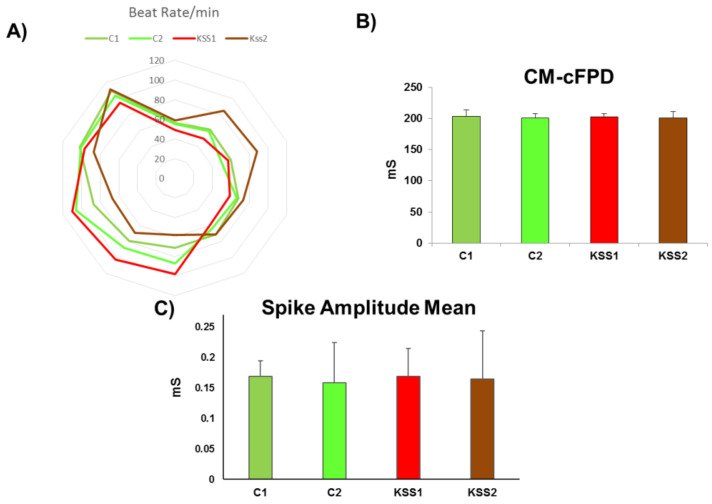
Cardiomyocyte functions were recorded using multi-electrode array. (**A**) The beat rates of the Kearns Sayre syndrome patients (KSS1 and KSS2) induced pluripotent stem cells (iPSC)-derived Cardiomyocytes (CM) did not show any difference from the iPSC derived CM of healthy controls (C1 and C2); (**B**) The corrected field potential of the iPSC derived CM of patients was not different from iPSC derived CM of healthy controls; (**C**) Spike amplitude mean did not differ between the control and KSS iPSC derived CM. *n* = 3, each experiment was repeated 3–4 times.

The most regular clinical presentation of the KSS patients is cardiac conduction blocks. Atrioventricular block is the most common cardiac conduction deficit observed in KSS patients, this often progresses to complete blockage of electrical conduction from atrium to ventricle. In the current study we measured beat rates and field potential of iPSC derived cardiomyocytes as a marker of cardiac contractility. There were no aberrations detected in the beat rates, corrected field potential and spike amplitude mean in CM of both the patients compared to iPSC derived CM from healthy individuals (Figure 6A–C).

## 4. Discussion

The diagnosis of hereditary mitochondrial disorders including KSS have improved steadily through advances in next generation sequencing, candidate gene sequencing, understanding of the metabolome, respiratory chain analysis and advances in computational biology. However, treatment options are lagging behind. The involvement of central nervous system and cardiac abnormalities are key manifestations in KSS patients. As the disease slowly progresses, prognosis for the affected individuals is determined by the number of organs involved, which increases with time. The underlying cause for the various organ systems manifesting at different times is often the energy deficit resulting from skewed heteroplasmy towards defective mtDNA. This energy crunch manifests as loss of cells and tissue function compromise. Functional organ or cellular replacement thus provides a promising avenue to stop or reverse the disease progression. In this regard, conventional options such as organ transplants are associated with multiple issues such as immunological barriers and scarcity of donors [26]. However, with the discovery of iPSC technology, patient specific personalized autologous cell-based therapies are possible without the complications related to immune rejection. Particularly in case of patients with KSS, mutations in mitochondrial DNA are largely found in the muscle tissue and are predominantly absent in the blood cells. Therefore, we conceptualized that PBMNC from patients with KSS can be reprogrammed to generate healthy and “mutation free” iPSC lines. These patient specific iPSC can be banked for future applications as isogenic source of cell replacement therapy to treat affected organs in KSS patients. 

Induced pluripotent stem cell technology is recently gaining ground in the field of disease modelling and drug development and has made it possible to produce pluripotent cells from diseased patients, exhibiting same genetic errors [27]. These patient derived iPSCs can be differentiated into any mature cell type in the body to mimic disease specific abnormal metabolic physiology and serve as a model for investigating disease mechanisms and drug testing [28]. Furthermore, the ability of iPSC to differentiate into any cell type in the body makes them excellent candidate cell type for cell replacement therapies. The embryonic stem (ES) cells as source of cell therapy are associated with several challenges including ethical concerns and immunogenicity of ES cells, as the ES cells are always derived from donors. However, iPSC can be easily created from the adult tissue of the patient and can be used as an autologous source of cell therapy. In fact, iPSC are already being tested in different pre-clinical models and in clinical trials for several diseases as an autologous source of cell replacement therapies [29]. However, the concept of reprogramming healthy cells from patients to generate “mutation free” isogenic patient specific iPSC lines has not been exploited yet. In the current proof of concept study, we reprogramed blood cells from two KSS patients and healthy individuals (controls). Our data demonstrate that iPSC and iPSC derived fibroblasts, NPC and CM did not show any mtDNA deletions which were only present in the mtDNA isolated from the patients’ muscle biopsies. A common 4.9 kb deletion is observed in KSS patients, which contains the MT-CO3 gene and detection of this common deletion fragment through conventional PCR would further validate our findings. However, the primers for qPCR in our studies were designed specifically to detect the gene encoding this MT-CO3 transcript. The MT-CO3 gene will get amplified and detected only if the DNA segment containing this gene is not deleted from the mtDNA. Such amplification was observed in both patients’ PBMNC and iPSC. Further, the reprogramming efficacy of PBMNC toward iPSC in KSS patient samples were comparable to PBMNC from healthy individuals. The quality of PBMNC derived iPSC was further assessed by investigation of several biochemical parameters. In KSS patients, the major energy defect detected is downregulation of ATP levels. Therefore, therapeutic interventions aimed at increasing ATP levels available to the cells have been beneficial in patients with inborn mitochondrial disorders including KSS [30]. Interestingly, in the current study, we found no difference in ATP synthesis in iPSC and iPSC derived fibroblasts, NPC and CM of KSS patients and healthy individuals. Therefore, mitochondrial energy producing machinery in patient PBMNC derived iPSC and differentiated cells was intact. Further, increased production of ROS and lactate levels in KSS patients is often reported. In the current study we found no change in ROS generation and lactate accumulation in iPSC and their derivatives from KSS patients and healthy individuals. The quality of mitochondria was further validated by the assessment of mitochondrial membrane potential in patient cells and in healthy controls. Mitochondrial membrane potential is a very good indicator of mitochondrial activity and health as it reflects the process of electron transport and oxidative phosphorylation and ATP production. In case of mitochondrial disorders including KSS, alterations in mitochondrial membrane potential are often reported. In the current study the mitochondrial membrane potential of patients derived iPSC, fibroblasts, NPC and cardiomyocytes were not different from cells of healthy individuals. This was a further verification of health of mitochondria as well as PBMNC derived iPSC. The cardiac abnormalities including cardiac conduction block, sudden cardiac death and syncope are regularly reported in KSS patients, evaluation of iPSC derived cardiomyocytes from both the KSS patients using multi-electrode array did not record any perceptible dysregulation in beat rate, spike amplitude (which provides height of action potential) and field potential. These findings provide further proof of normal physiology of patients derived iPSC and differentiated cells.

Therefore, in the current study, peripheral blood mononuclear cells derived iPSC represented an isogenic source of patient-specific personalized cell replacement therapies, which need not undergo any genetic modulation. This useful feature circumvents the complexities which accompany prospective gene-correction therapies or only mitochondrial replacement therapies. Further, the iPSC and their multiple derivatives were found to be equivalent in their physiological components to healthy iPSC and derivatives. Our KSS patients’ PBMNC derived cells did not display any clinical or biochemical aberrations that are clinically evident in the patients. Thus, the heteroplasmy of mtDNA mutation expression within the specific tissues can be positively exploited to design patient-specific treatment modalities for KSS patients.

## Data Availability

The data presented in this study are available in this article.

## Supplementary Materials

The following are available online at https://www.mdpi.com/2073-4409/10/3/568/s1, Figure S1: Karyotypic abnormalities in iPSC were investigated at intervals of 20–25 passages using a commercial kit (StemCell Technologies); Figure S2. Assessment of biochemical parameters in iPSC, fibroblasts (Fibs), neural progenitor cells (NPC) and cardiomyocytes (CM) in healthy control (Cntrl), KSS1 and Leigh Syndrome (LS) patients; Table S1: List of primers used in the study.
