# Supplementary material for: Generation and Evaluation of Isogenic iPSC as a Source of Cell Replacement Therapies in Patients with Kearns Sayre Syndrome
Source: Cells. 2021 Mar 5;10(3):568. doi: 10.3390/cells10030568 (PMC7998189; doi:10.3390/cells10030568)
Supplement: Supplementary file 1 [file cells-10-00568-s001.pdf]

cells

MDPI

Supplementary materials

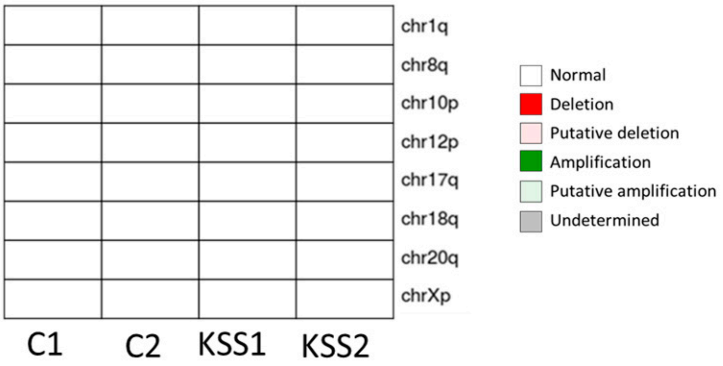

Gene	Description
Control 1	All tested loci are normal.
Control 2	All tested loci are normal.
KSS1	All tested loci are normal.
KSS2	All tested loci are normal.

Supplementary Figure S1. Karyotypic abnormalities in induced pluripotent stem cells (iPSC) were investigated at intervals of 20–25 passages using a commercial kit (StemCell Technologies, Vancouver, BC, Canada). None of the iPSC lines generated in the study reported any karyotypic abnormalities.

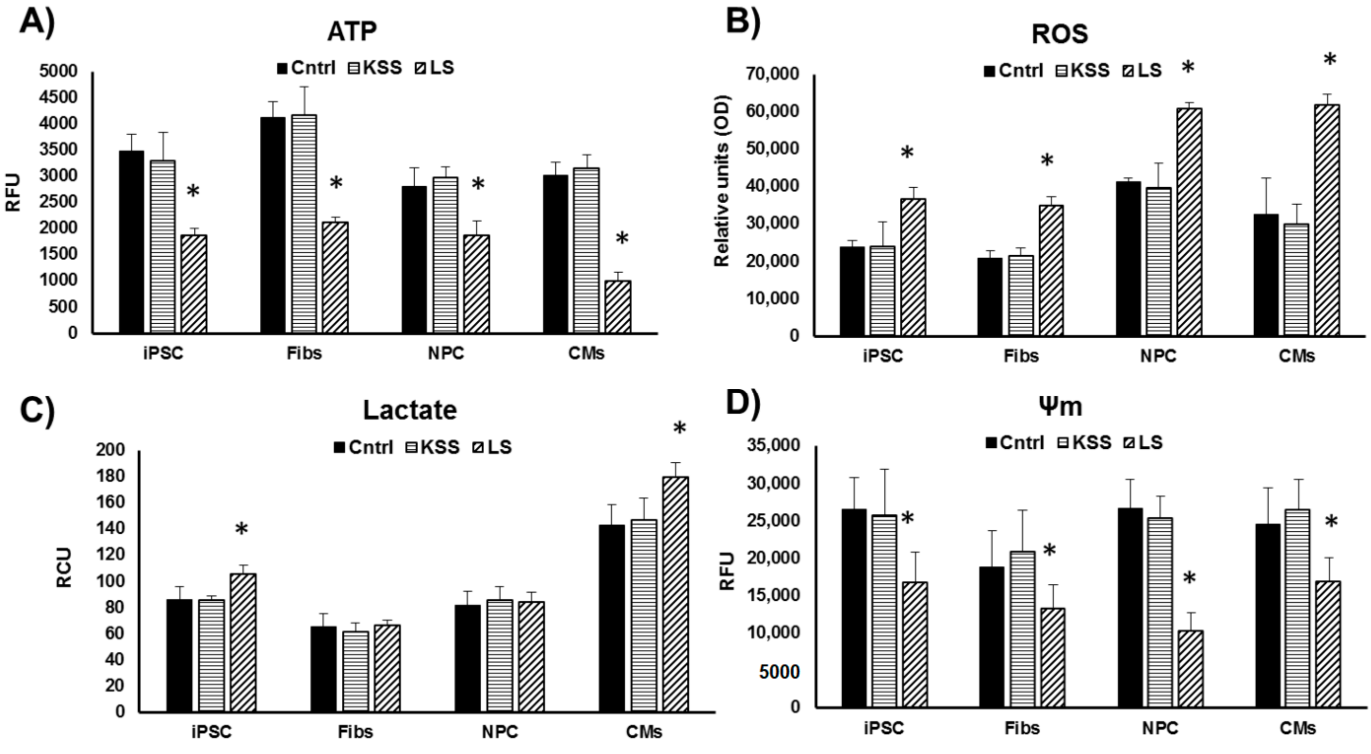

Supplementary Figure S2. Assessment of biochemical parameters in induced pluripotent stem cells (iPSC), fibroblasts (Fibs), neural progenitor cells (NPC) and cardiomyocytes (CM) in healthy control (Cntrl), Kearns Sayre syndrome patient (KSS) and Leigh Syndrome (LS) patient. (**A**) ATP generation in healthy control, KSS and LS cells; (**B**) ROS generation in

healthy control, KSS and LS cells; (**C**) Lactate levels in control, KSS and LS cells; (**D**) Mitochondrial membrane potential in control, KSS and LS cells. $n = 3$. $*p < 0.05$ in comparison to control.

Supplementary Table S1. List of primers used in the study.

Gene	Forward	Reverse
POU5F1	GGAAGGAATTGGGAACACAAAGG	AACTTCACCTTCCCTCCAACCA
Tra-1-60	CAACCCGGCCCAAGATAAGT	GGCAGGGAGCTTAGTGTGAA
Sox17	GAGCCAAGGGCGAGTCCCGTA	CCTTCCACGACTTGCCCAGCAT
Foxa2	CCCCTGAGTTGGCGGTGGT	TTGCTCACGGAAGAGTAG
Brachury T	ACCCAGTTCATAGCGGTGAC	CCATTGGGAGTACCCAGGTT
NCam1	AACAAAGCATGATGGGTGAA	GTCTGTGGTGTTGGAAATGC
OTX2	CAAAGTGAGACCTGCCAAAAAGA	TGGACAAGGGATCTGACAGTG
Pax6	ATGGGCGGAGTTATGATACCTAC	GGAACTTGAACTGGAACTGACA
RPLPO	CACTGGCTGAAAAGGTCAAGG	GACTTGGTGTGAGGGGCTTA
HPRT1	CACGGCTGTGCTAGTTCAGTA	TCGACAAGCCCAGAAACTTGT
RNR2 (16SrRNA)	TTTACGACCTCGATGTTGGATCAG	CCTTTCGTACAGGGAGGAATTTG
MT-CO3	TTTCCGACGGCATCTACGG	TACAAAATGCCAGTATCAGGCG

Copyright: © 2021 by the authors. Licensee MDPI, Basel, Switzerland. This article is an open access article distributed under the terms and conditions of the Creative Commons Attribution (CC BY) license (http://creativecommons.org/licenses/by/4.0/).
